# Physical activity levels, duration pattern and adherence to WHO recommendations in German adults

**DOI:** 10.1371/journal.pone.0172503

**Published:** 2017-02-28

**Authors:** Agnes Luzak, Margit Heier, Barbara Thorand, Michael Laxy, Dennis Nowak, Annette Peters, Holger Schulz, for the KORA-Study Group

**Affiliations:** 1 Institute of Epidemiology I, Helmholtz Zentrum München - German Research Center for Environmental Health, Neuherberg, Germany; 2 Institute of Epidemiology II, Helmholtz Zentrum München - German Research Center for Environmental Health, Neuherberg, Germany; 3 MONICA/KORA Myocardial Infarction Registry, Central Hospital of Augsburg, Augsburg, Germany; 4 Institute of Health Economics and Health Care Management, Helmholtz Zentrum München - German Research Center for Environmental Health, Neuherberg, Germany; 5 Institute and Outpatient Clinic for Occupational, Social and Environmental Medicine, University Hospital of Munich (LMU), Munich, Germany; 6 Comprehensive Pneumology Center Munich (CPC-M), Member of the German Center for Lung Research, Munich, Germany; Universidad Europea de Madrid, SPAIN

## Abstract

**Background:**

Intensity and duration of physical activity are associated with the achievement of health benefits. Our aim was to characterize physical activity behavior in terms of intensity, duration pattern, and adherence to the WHO physical activity recommendations in a population-based sample of adults from southern Germany. Further, we investigated associations between physical activity and sex, age, and body mass index (BMI), considering also common chronic diseases.

**Methods:**

We analyzed 475 subjects (47% males, mean age 58 years, range 48–68 years) who wore ActiGraph accelerometers for up to seven days. Measured accelerations per minute obtained from the vertical axis (uniaxial) and the vector magnitude of all three axes (triaxial) were classified as sedentary, light or moderate-to-vigorous physical activity (MVPA) according to predefined acceleration count cut-offs. The average minutes/day spent in each activity level per subject served as outcome. Associations of sex, age, BMI, and seven chronic diseases or health limitations, with the activity levels were analyzed by negative binomial regression.

**Results:**

Most of the wear time was spent in sedentarism (median 61%/day), whereas the median time spent in MVPA was only 3%, with men achieving more MVPA than women (35 vs. 28 minutes/day, p<0.05). Almost two thirds of MVPA was achieved in short bouts of less than 5 minutes, and 35% of the subjects did not achieve a single 10-minute bout. Hence, only 14% adhered to the WHO recommendation of 2.5 hours of MVPA/week in at least 10-minute bouts. Females, older subjects and obese subjects spent less time in MVPA (p<0.05), but no clear association with hypertension, asthma, diabetes, chronic obstructive pulmonary disease, anxiety/depression, pain or walking difficulties was observed in regression analyses with MVPA as outcome.

**Conclusions:**

Activity behavior among middle-aged German adults was highly insufficient, indicating a further need for physical activity promotion in order to gain health benefits.

## Background

Being physically active is linked to several health benefits, including the improvement of functional ability, cardiorespiratory fitness, metabolic health, and the prevention of premature death [1–4]. Although every bit of physical activity (PA) counts towards health benefits, the frequency, intensity and duration have great impact on these benefits [1–3]. Existing evidence supports a dose-response relationship of PA and reduced risk of chronic conditions like type 2 diabetes, cardiovascular disease, or stroke [3]. Therefore, the World Health Organization (WHO) recommends that adults without mobility-related chronic diseases should accumulate at least 2.5 hours of moderate-intensity PA per week spent in bouts of at least 10 minutes [1].

In order to improve adherence and to establish PA promotion, it is important to collect evidence on the pattern of PA behavior, however assessing PA is challenging [5–7]. Various approaches exist that rely either on self-reports or objective motion sensors [5, 6]. A systematic review comparing self-reported and objectively measured activity in adults found that correlations were generally low-to-moderate [6]. This is illustrated by the results of the fitness health survey for England in 2008 in which, based on self-report, 39% of men and 29% of women aged >16 years achieved at least moderate activity for a minimum of 30 minutes at five or more days a week; while, based on accelerometer data, only 6% of men and 4% of women met this threshold in at least 10 minute bouts [8]. Self-report tends to overestimate the time spent physically active at high intensity levels, whereas activity gained through lifestyle activities, e.g. during house work or active transportation, might be underestimated [6, 9]. Accelerometry provides the possibility to measure PA in a more standardized manner regardless of the current fitness level that might influence the subjectively perceived and reported intensity of PA [6, 9, 10]. Therefore, the number of studies that investigate objectively measured PA is increasing [11]. However, to date few population-based studies that objectively investigate the adherence to PA recommendations for moderate to vigorous activity (MVPA) in adults exist [8, 9, 12–14]. In the United States, data from the National Health and Nutrition Examination Survey (NHANES) revealed that if at least 30 minutes of MVPA on 5 out of 7 days, in bouts of at least 8–10 minutes, was considered as the recommended threshold, the adherence was less than 5% among adults [9]. Further, European data for adults from England, Norway, Sweden and Portugal revealed a generally low achievement of PA recommendations (1% to 20%) [8, 12–14].

Large scale, population-based data on PA in German adults was provided from the health monitoring system at the Robert Koch Institute based only on self-reports. Among 7671 participants, aged 18–79 years, 20% reported to achieve the WHO threshold of at least 2.5 hours of MVPA/week [15]. Furthermore, investigations of objectively measured PA in Germany were performed in 168 participants aged 65–89 years. Of those, 12% reached 2.5 hours of MVPA/week in bouts lasting at least 10 minutes [16]. To our knowledge further data on PA from population-based studies assessing PA with accelerometers in German adults are missing.

The aim of the present study was to determine the PA levels and PA duration pattern assessed by accelerometry in a German adult cohort aged 48–68 years. Duration patterns of interest were the time spent in MVPA assessed in various bouts and the adherence to the WHO PA recommendations for adults categorized by sex and age. A further goal was to analyze the associations of sex, age, and BMI with activity levels serving as outcome. Since evidence indicates that subjects with chronic health issues engage less in PA [16, 17], we further addressed, if common chronic diseases or health limitations such as hypertension, asthma or physical complaints were associated with PA in a population-based sample of German adults.

## Methods

### Study population

**Fig 1:**
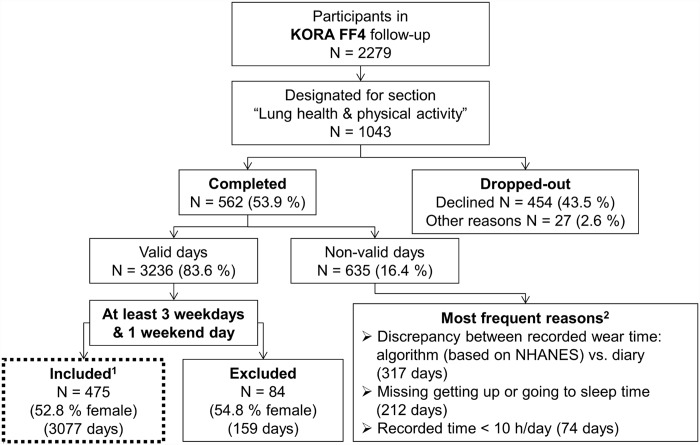
Participant recruitment and data handling. ^1^After exclusion of 3 outliers. ^2^Multiple reasons possible (detailed description in S1 Methods). NHANES algorithm: National Health and Nutrition Examination Survey algorithm [19].

The present analysis was based on a follow-up study of the KORA (Cooperative Health Research in the Region of Augsburg) S4 cohort comprising 4261 adults examined in 1999–2001. The primary study design has been described previously [18]. Between June 2013 and September 2014, 2279 subjects (age range 38 to 88 years) participated in the KORA FF4 follow-up of whom 1043 were designated to participate in the “Lung health & physical activity” section, which involved spirometric lung function measurements and PA assessment by accelerometry (Fig 1). The study was approved by the responsible ethics committee of the Bavarian Medical Association. The investigations were carried out in accordance with the Declaration of Helsinki and written informed consent was obtained from all participants.

Baseline information on sociodemographic variables, self-reported physician diagnosis of common chronic diseases such as asthma or diabetes, and medication use was obtained during a standardized interview, also including questionnaires such as the EuroQol—5 Dimensions (EQ-5D) questionnaire and the shortened medical outcome survey (SF-12) which are assessment tools for health status and health-related quality of life. Further, participants underwent a standardized medical examination. Height and weight were assessed while subjects were wearing light clothes without shoes. After a rest of at least 5 minutes in a sitting position, blood pressure was measured three times on the right arm and determined by the mean of the second and third measurement.

In the present analysis, diabetes was defined based on self-reported physician diagnosis or use of antidiabetic agents. Hypertension was defined as blood pressure greater or equal to 140/90 mmHg or if the subject reported the use of antihypertensive medication given that the subject was aware of being hypertensive. Subjects who reported a doctor’s diagnosis of chronic bronchitis or chronic obstructive pulmonary disease (COPD) were classified as having COPD. Information on mobility, pain/physical complaints and anxiety/depression was derived from the EQ-5D, and on overall health from the SF-12 questionnaire.

### Physical activity assessment

The subjects received the accelerometer, instructions on its use, and a diary sheet at the study center. Participants were asked to start wearing the monitor on the following morning immediately after getting out of bed, and to complete a daily diary (S1 Methods) which included the time of getting up, going to work, getting back from work, start and end of specific sport episodes, type of sport performed, and going to sleep. Non-wear time was also included along with an explanation e. g. taking a shower or swimming. The measurement of PA was obtained from ActiGraph GT3X (Pensacola, Florida) accelerometers with the use of the ActiLife software (version 6.11.2, firmware 4.4.0). The accelerometer was attached to an elastic belt and worn over a one week period at the hip on the side of the dominant hand.

Measured accelerations primarily sampled at 30 Hz and stored at 1 Hz were resampled in one minute epochs for further data analysis. As recommended by ActiGraph, data filtering was set to default (‘normal’). 1-minute epochs were chosen to allow for comparisons with other population-based studies [8, 9, 12–14] and for applicability of available activity level calibration studies that were performed based on accelerometer counts and oxygen consumption per minute [20, 21]. Since it is debated whether the triaxial assessment of PA might be able to capture more complex movement than uniaxial and therefore might derive a better estimation of PA, we used both information for our analysis [22, 23].

According to the recorded acceleration value per minute, each minute was classified into one of three activity levels (sedentary, light, MVPA) using two approaches: the uniaxial count cut-offs of the vertical axis were based on the commonly used Freedson et al. [20] cut-offs and the triaxial vector magnitude cut-offs referred to Sasaki et al. [21]. In our analysis the uniaxial cut-offs for sedentary, light and MVPA were set to ≤100, >100, and ≥1952 counts/minute and the triaxial cut-offs to <200, ≤2690, and >2690 counts/minute, respectively. Further, the minutes per person obtained in each single activity level were summed and divided by the number of recorded days; resulting in the average minutes per day per subject spent in sedentary, light, or MVPA. Several steps were applied to assure high quality data, which are described in detail in the additional file–S1 Methods. Days were excluded if the difference between the non-wear time algorithm, which was based on the NHANES algorithm [19], and the diary non-wear time was >120 minutes (if the accelerometer indicated a non-wear time) or >60 minutes (if the non-wear time was reported in the diary). Further reasons for exclusion were missing information on time spent awake, no reported non-wear time over the whole reporting period, and an incorrect handling of the accelerometer. MVPA for non-wear time <2 hours during sport was imputed using the percent of time spent in MVPA during PA of each subject or, if not available, through sex specific averages [24]. Through imputation, a mean of 3 minutes of MVPA/day (about 11%) was added to the recorded MVPA of 56 subjects. Valid days were required to have at least 10 hours of recording time, or >7 hours if the subjects reported day length was <10 hours. To account for differences in PA between weekdays and weekends that were shown in previous studies [25, 26], subjects were included in the analysis only if they had at least 3 valid weekdays and 1 valid weekend day.

### Statistical analyses

Pearson’s Chi-square test, Fishers exact test (if cell count <5), and Wilcoxon rank-sum test (adjusting for multiple testing using the Holm correction, if necessary) were used to assess sex specific differences, group differences in the analyzed study population, and differences between subjects designated to participate in the “Lung health & physical activity” section and those who participated in the PA assessment. Time counts of all three activity levels were averaged over the reporting period for each subject and described by median and the 25^th^ and 75^th^ percentiles.

Among the study population, age, ranging from 48 to 68 years, was grouped into three age tertiles (low: <55 years; intermediate: 55–61, and advanced: >61 years). BMI (kg/m^2^) was calculated using values of height and weight obtained by physical examination in the study center. Subjects were categorized as normal weight (BMI 18–25 kg/m^2^), overweight (BMI ≥25 kg/m^2^), and obese (BMI ≥30 kg/m^2^) [27].

Mean minutes of MVPA/subject showed a skewed distribution, with a great variance. Negative binomial regression, which accounts for overdispersion, was applied to investigate the associations between time spent in each PA level (sedentary, light, or MVPA) with sex, age and BMI.

Sedentary, light and MVPA served as outcome in all analyses. Each outcome was expressed in minutes averaged over the recording period for each subject and rounded to the nearest integer. For each outcome, a basic model was applied, which was adjusted for sex, age and BMI. Stepwise selection (combined forward and backward selection), using the lowest Akaike information criterion (AIC) as stopping rule, was performed to determine a model for each activity level considering several covariates. Besides sex, age and BMI, the following covariates were assessed: (1) season, categorized as winter (start of measurement: December to February), spring (March to May), summer (June to August), and autumn (September to November), (2) education, categorized as low (<10 years of school), middle (10 years of school) and high (>10 years of school), (3) hypertension, (4) diabetes, (5) asthma, (6) COPD, (7) difficulties with walking, (8) pain or physical complaints, and (9) anxiety or depression. All models with sedentary and light activity as outcome were additionally adjusted for average wear time/day. Our study population comprised Caucasians only, so ethnicity was not considered as a covariate.

A potential interaction effect of sex was tested in the final models. Sensitivity analyses were performed through exclusion of subjects reporting fair/poor overall health (N = 49) and exclusion of subjects who reported myocardial infarction (N = 14) and/or stroke (N = 8), to avoid possible bias towards a lower PA due to these diseases. All analyses were performed with uniaxial cut-offs. To assess the impact of uni- vs. triaxial motion monitoring and to ensure better comparability to other studies, we replicated all aforementioned analyses using triaxial cut-offs.

Adherence to the WHO PA recommendation was defined as achieving at least 2.5 hours of MVPA/week in bouts of at least 10 minutes. Therefore, hours of MVPA/day spent in bouts of at least 10 minutes were averaged across the recording period for each subject and multiplied by seven.

For all analyses the statistical program R, version 3.2.0 [28], was used and p-values below 0.05 were considered statistically significant.

## Results

Of 562 subjects who provided accelerometer data, 478 had valid data that passed the quality control (Fig 1, S1 Methods). Three subjects were excluded from the analyses; one male due to extremely high total measured uniaxial MVPA values (averaged 223 minutes/day compared to the median 35 minutes/day in all males), one female due to extremely high MVPA values in the triaxial measurement (averaged 252 minutes/day compared to the median 43 minutes/day in all females) and a second male due to severe obesity (BMI of 62 kg/m^2^). The final study population comprised of 475 subjects with a mean age of 58 years (Table 1). At least 6 valid days were recorded by 89% of the subjects while 2% had 4 valid days. Males accounted for 47% of the subjects. The prevalence of overweight and obesity was 80% in males and 63% in females, respectively, and differed significantly between sexes. Subjectively reported overall health was good to excellent in 90% of subjects. 53% reported at least one chronic morbidity when considering hypertension, diabetes, asthma, COPD, walking difficulties, pain/physical complaints or anxiety/depression. In comparison to all other participants designated to the “Lung health & physical activity” section of the KORA FF4 follow up (N = 481) participants in PA (N = 562) reported more often a very good overall health. All other population characteristics did not differ (Table A in S1 Tables).

**Table 1:** Characteristics of the study population (% (N)).

	Males	Females	Total
	47.2% (224)	52.8% (251)	100% (475)
**Age**, years (mean: 58; range: 48–68)			
<55	34.8 (78)	30.7 (77)	32.6 (155)
55–61	31.7 (71)	38.6 (97)	35.4 (168)
>61	33.5 (75)	30.7 (77)	32.0 (152)
**Working**, yes*	75.9 (170)	64.9 (163)	70.1 (333)
**Education**			
Low (<10 years of school)	46.0 (103)	45.8 (115)	45.9 (218)
Medium (= 10 years of school)	25.0 (56)	32.7 (82)	29.1 (138)
High (>10 years of school)	29.0 (65)	21.5 (54)	25.1 (119)
**Body mass index,** kg/m^2^*			
Normal (<25)	19.6 (44)	37.1 (93)	28.8 (137)
Overweight (≥25, <30)	50.9 (114)	35.5 (89)	42.7 (203)
Obese (≥30)	29.5 (66)	27.5 (69)	28.4 (135)
**Overall health** (reported)			
Excellent/very good	32.1 (72)	25.1 (63)	28.4 (135)
Good	60.3 (135)	62.2 (156)	61.3 (291)
Fair/poor	7.6 (17)	12.7 (32)	10.3 (49)
**Hypertension**, yes*	39.3 (88)	29.1 (73)	33.9 (161)
**Diabetes**, yes	6.2 (14)	7.2 (18)	6.7 (32)
**Asthma ever**, yes	8.0 (18)	11.6 (29)	9.9 (47)
**COPD**, yes	7.1 (16)	10.4 (26)	8.8 (42)
**Difficulties in walking**			
Not at all/slight	95.1 (213)	93.2 (232)	94.1 (445)
Moderate/hard	4.9 (11)	6.8 (17)	5.9 (28)
Pain or physical complaints*			
Not at all/slight	90.2 (202)	81.3 (204)	85.5 (406)
Moderate/hard	9.8 (22)	18.7 (47)	14.5 (69)
Feeling anxious/depressed*			
Not at all/slight	96.9 (216)	90.0 (226)	93.2 (442)
Moderate/strong	3.1 (7)	10.0 (25)	6.8 (32)

*p<0.05 in Chi-square test (males vs. females).

COPD: Chronic obstructive pulmonary disease.

### Activity levels

**Fig 2:**
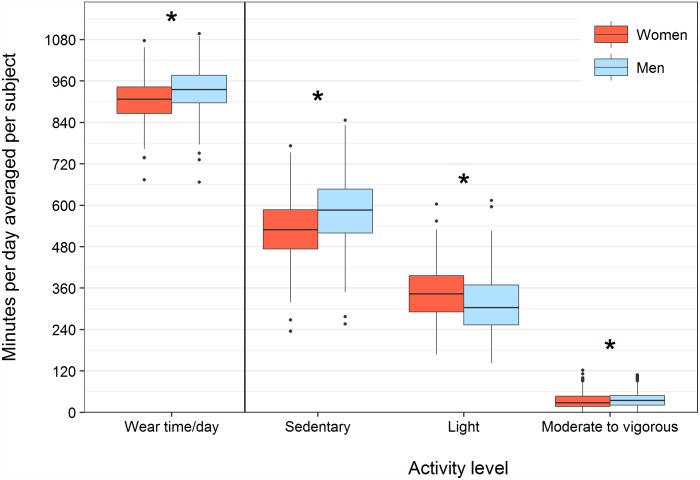
Total wear time and time spent in sedentary, light and moderate to vigorous (MVPA) activity in minutes/day averaged per subject. *p<0.05 in Wilcoxon rank-sum test (males vs. females).

The overall median recorded wear time averaged per subject was about 15 h/day (Fig 2). The daily median time spent sedentary was higher in males than in females (586 vs. 529 minutes, respectively, p<0.05), whereas females engaged in more light activity (303 vs. 343 minutes, respectively, p<0.05) (Fig 2, Table B in S1 Tables). The daily median time spent in MVPA was 35 minutes (25-75^th^ percentile: 21–49 minutes) for males and 28 minutes for females (25-75^th^ percentile: 17–47 minutes) (p<0.05). In the total population, of the daily recorded time, a median of 61% (25-75^th^ percentile: 54–67%) was spent in sedentary behavior, whereas the median time spent in light and MVPA was 35% (25-75^th^ percentile: 30–42%) and 3% (25-75^th^ percentile: 2–5%), respectively.

Age comparisons revealed that subjects aged <55 years spent more minutes in MVPA/day than those aged >61 years, irrespective of sex (median: 35 and 26 minutes in males, and 31 and 21 minutes in females, respectively, p<0.05). Normal and overweight men and women spent more time in MVPA/day compared to obese subjects (median: 44, 36 and 28 minutes in males, and 34, 28, and 20 minutes in females, respectively (p<0.05); Table C in S1 Tables).

### Investigation of covariates

Whilst in sedentary and light activity no associations were seen with age and BMI in the basic models, negative associations were present between MVPA and the highest age tertile (ratio 0.75; 95% confidence interval (CI) 0.65, 0.87)) as well as obesity (ratio 0.67; CI: 0.57, 0.78) (Table 2). Exclusion of subjects with myocardial infarction and/or stroke (N = 21) did not substantially alter the results. Models with the lowest AIC after stepwise selection (combined forward and backward selection) considering sex, age, BMI, seven chronic morbidities or health limitations, season and education as covariates are shown in Table 2. Associations of sex, the highest age tertile and obesity with MVPA remained significant.

**Table 2:** Count ratios (95% confidence intervals) of uniaxial average minutes/day of moderate-to-vigorous physical activity (MVPA), sedentary or light activity estimated by negative binomial regression.

Outcome		Sedentary		Light		MVPA	
Variables	Model	Basic^1^	Stepwise^1^	Basic^1^	Stepwise^1^	Basic	Stepwise
**Sex**	Female	-	-	-	-	-	-
	Male	**1.06 (1.03; 1.10)**	**1.06 (1.03; 1.09)**	**0.88 (0.84; 0.92)**	**0.89 (0.85; 0.93)**	**1.24 (1.10; 1.39)**	**1.23 (1.09; 1.38)**
**Age, years**	<55	-	-	-	-	-	-
	55–61	0.97 (0.94; 1.01)	**0.97 (0.93; 1.00)**	1.05 (1.00; 1.11)	**1.06 (1.00; 1.11)**	0.99 (0.86; 1.13)	0.99 (0.86; 1.14)
	>61	1.04 (1.00; 1.07)	**1.04 (1.00; 1.08)**	0.97 (0.91; 1.02)	0.96 (0.91; 1.01)	**0.75 (0.65; 0.87)**	**0.76 (0.65; 0.87)**
**BMI (kg/m**^**2**^**)**	Normal (<25)	-	-	-	-	-	-
	Overweight (≥25, <30)	0.99 (0.96; 1.03)	1.00 (0.97; 1.04)	1.02 (0.97; 1.08)	^x^	**0.85 (0.74; 0.98)**	0.87 (0.76; 1.00)
	Obese (≥30)	1.03 (0.99; 1.07)	1.04 (1.00; 1.08)	0.99 (0.94; 1.05)	^x^	**0.67 (0.57; 0.78)**	**0.71 (0.60; 0.83)**
**Education**	Low (<10 years of school)	-	-	-	-	-	-
	Medium (= 10 years of school)	-	**1.05 (1.01; 1.08)**	-	**0.93 (0.88; 0.98)**	-	^x^
	High (>10 years of school)	-	**1.09 (1.06; 1.13)**	-	**0.87 (0.82; 0.92)**	-	^x^
**Pain/physical complaints**	Not at all/slight	-	-	-	-	-	-
	Moderate/hard	-	^x^	-	^x^	-	**0.83 (0.70; 0.98)**
**Hypertension**	No	-	-	-	-	-	-
	Yes	-	^x^	-	^x^	-	0.91 (0.80; 1.04)
**Diabetes**	No	-	-	-	-	-	^-^
	Yes	-	**1.07 (1.01; 1.13)**	-	**0.89 (0.82; 0.97)**	-	^x^
			[stratified by sex: **1.12 (1.04; 1.21)** females; 0.99 (0.91;1.08) males]		[stratified by sex: **0.80 (0.72; 0.88)** females; 1.00 (0.87;1.16) males]		

Significant associations (p<0.05) are shown in bold. Basic model was adjusted for sex, age, and body mass index (BMI). Besides sex, age, and BMI, considered variables in the stepwise selection model (combined forward and backward selection) were: season, education, hypertension, diabetes, asthma, chronic obstructive pulmonary disease (COPD), difficulties with walking, pain or physical complaints, and anxiety/depression. Models with the lowest Akaike information criterion (AIC) are shown.

^1^Model additionally adjusted for average recorded wear time/day.

^x^Covariate did not remain in main model.

In our cohort, no or only unstable associations of morbidity with PA levels could be determined. Pain or physical complaints were negatively associated with MVPA in the best fit model obtained by stepwise selection, but were not significant after exclusion of subjects with myocardial infarction and those with stroke (p<0.1). Results were only slightly altered after exclusion of subjects who reported fair/poor overall health. Due to an interaction effect between sex and diabetes, we stratified the models of sedentary and light activity by sex. After stratification, diabetes showed a negative association with light activity and a positive association with time spent sedentary in females only (p<0.05; Table 2). Further, an unstable interaction effect was found between sex and the highest age tertile (>61 years), suggesting a positive association of advanced age with sedentary and a negative association with light activity in men, while no association was shown in females (data not shown).

### Duration pattern of time spent in MVPA

**Fig 3:**
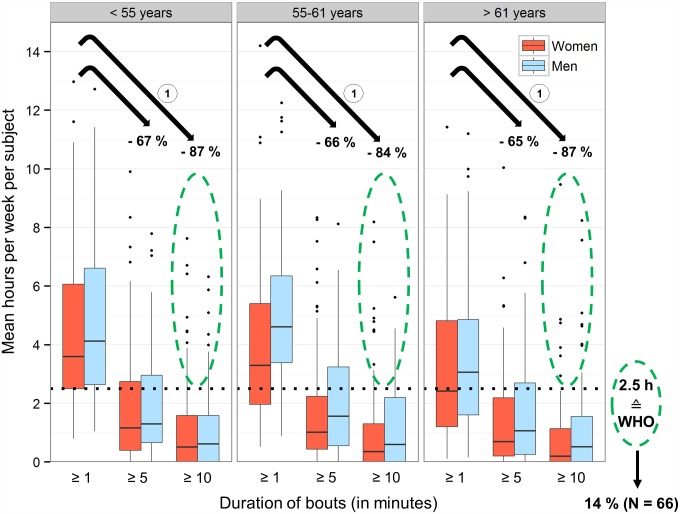
Time spent in moderate to vigorous activity (MVPA) in at least 1, 5, and 10 minute bouts categorized by age and adherence to WHO physical activity recommendations. Hours/day spent in MVPA are averaged over the recording period of each subject and multiplied by seven. MVPA cut-off set at 1952 counts/minute (uniaxial). Green dashed circles: Subjects who met the WHO physical activity recommendation of ≥ 2.5 hours of MVPA/week in at least 10 minute bouts. ^**1**^ Median percent decrease in time spent in MVPA after excluding bouts shorter than 5 and 10 minutes, respectively.

The majority of time spent in MVPA was accumulated in short bouts i.e. less than 5 minutes (Fig 3, Table D in S1 Tables). Overall, the median time spent in bouts of at least 1 minute was 3.52 hours/week, 1.11 hours in bouts of at least 5 minutes and only 0.45 hours in bouts of at least 10 minutes. The median decrease of time spent in MVPA per subject lasting at least 1 minute was 66% when the bout was raised to at least 5 minutes and 86% when the bout was further raised to at least 10 minutes. MVPA bouts of at least 10 minutes did not differ between age tertiles for either sex.

We found that 168 subjects in our population did not achieve a single episode of MVPA lasting at least 10 minutes. While 26% of normal weight subjects did not reach at least one episode of MVPA spent in at least 10 minutes, this amount was almost double in obese subjects (45%) (Table E in S1 Tables). The highest proportion of subjects who did not meet this 10 minute threshold was found among those with difficulties in walking or pain/physical complaints.

### Adherence to WHO physical activity recommendation

As recommended by the WHO adults should accumulate a minimum of 2.5 hours of at least moderate intensity PA/week spent in bouts of 10 minutes or greater. In our population, only 14% met this recommendation; 36 men and 30 women, respectively. While 15% of subjects aged <55 years adhered to the WHO recommendation, 11% reached this threshold in the advanced age category (>61 years). The highest prevalence of adherence was found within the middle aged group with 55 to 61 years (16%). Among subjects adhering to the WHO recommendation the prevalence of having hypertension, diabetes, asthma, COPD, walking difficulties, pain/physical complaints or anxiety/depression ranged from 3% (walking difficulties) up to 24% (hypertension) (Table E in S1 Tables), while 50% presented none of these morbidities.

### Comparison of triaxial to uniaxial results

When applying triaxial cut-offs, subjects were less inactive and spent more time in light activity or MVPA compared to uniaxial cut-offs (median 469 vs. 552 minutes sedentary, 397 vs. 324 minutes light, and 46 vs. 30 minutes MVPA, respectively), whereas group comparisons considering sex, age and BMI showed similar trends. Males, younger subjects and subjects with a lower BMI spent more time in MVPA (Tables B and C in S1 Tables). As observed for uniaxial data, the strongest associations in regression analyses were also present between MVPA and advanced age (ratio 0.77; CI: 0.67, 0.87) or obesity (ratio 0.79; CI: 0.69, 0.91) (Table F in S1 Tables). Obesity was negatively associated with light activity (ratio 0.91; CI: 0.86, 0.96) and positively with sedentary behavior (ratio 1.11; CI: 1.05, 1.16). Considering 1 minute, 5 minute and 10 minute bouts of time spent in MVPA showed a similar pattern as with uniaxial cut-offs (S1 Fig). In line with increased time spent in MVPA observed by triaxial assessment the number of subjects meeting the WHO recommendation increased from 66 to 81. Comparable to uniaxial, the adherence was 17.1% (41 males and 40 females) (Table D in S1 Tables), with 18% of subjects aged <55 years, 19% aged 55–61 years and 14% aged >61 meeting the threshold. Among subjects not achieving at least one episode of time spent in MVPA in a 10 minute bout, the highest prevalence of the investigated morbidities was still present among subjects with difficulties in walking (Table G in S1 Tables).

## Discussion

The time spent in MVPA was generally low among adults aged 48–68 years from southern Germany, while men engaged more in MVPA than women. MVPA was significantly lower among obese subjects and those aged >61 years. Only a median of 14% of MVPA was accumulated in bouts of at least 10 minutes, which would count towards meeting the WHO PA recommendations. This is why only 66 subjects (14%) met the threshold of at least 2.5 hours of MVPA/week in bouts of at least 10 minutes.

The low prevalence of adherence to the WHO PA recommendation is comparable to other European studies. In Norway, 20% of the study population (N = 3267), aged 20–85 years, accumulated at least 30 minutes of daily MVPA in bouts of 8–10 minutes [12]. Of 1114 Swedish participants (age range 18–79 years), 52% accumulated at least 30 minutes/day of MVPA, but only 1% reached 30 minutes/day in ≥ 3 periods of 10 minute bouts [13]. In Portugal, 7–9% participants aged 40–64 years and 3% aged 65 years or older, accumulated at least 30 minutes MVPA/day in periods of at least 10 minutes [14]. Similar to our study, all three studies used ActiGraph accelerometers, which is a commonly used device for objective PA assessment [11], but varied slightly in terms of cut-offs (1952 to 2020 counts/minute for MVPA), number of measured days (4 to 7) as well as application of international PA recommendations (e.g. allowing 1–2 minutes of non-movement throughout the 10 minute bout) and population characteristics such as age. Nevertheless, in general the adherence to the WHO PA recommendation among all studies was widely below 50%.

In our analysis, in both, men and women, the majority of wear time was spent sedentary, being higher in males than in females. A meta-analysis has recently reported that the risk for all-cause mortality is increased for subjects with high amounts of sedentary behavior, assessed as sitting time, but that this effect seems to be mitigated through PA [29]. Subjects in the highest quartile of PA and sitting time for >8h showed a lower mortality risk than those in the least active quartile and <4h sitting time. The highly active subjects achieved about 60–75 minutes moderate PA/day which was even higher than the recommended WHO threshold [29]. In contrast, not meeting the WHO PA recommendations was found to account for about 6% of the burden of coronary heart disease, 7% of type 2 diabetes, and 10% of colon cancers worldwide, which would be addressed by increasing PA [30].

German large scale population-based data, based on self-reports, reported an adherence to WHO PA recommendation of 18%, both in the 50–59 years and the 60–69 years age groups [15]. This prevalence was only slightly higher than the prevalence found in our cohort (14% uniaxial and 17% triaxial). However, despite the similar trend, no direct comparisons are possible, as the correlation between self-reported and directly measured PA was found to be only low-to-moderate [6].

Our data showed that the median number of daily minutes of MVPA decreased across age and BMI categories, and further, that males achieved more minutes of MVPA, which is in line with other studies [8, 9, 13, 14]. A similar pattern was shown in the health survey for England in 2008, where participants in the normal weight category spent on average fewer minutes in sedentary time and more time in MVPA than those who were in the overweight or obese category, showing also a declining gradient of averaged minutes spent in MVPA with age [8]. In contrast, we found that within all three age tertiles (<55, 55–61 and >61 years) the median decrease of averaged hours spent in MVPA/week per subject was around 66% when raising the consecutive MVPA counts to 5 minutes, and around 87% when raising this threshold to at least 10 minutes. While the highest amount of subjects not achieving a single MVPA bout of at least 10 minutes was seen among subjects reporting difficulties in walking (61%) and pain or physical complaints (58%), the majority of subjects reporting any other investigated morbidity reached at least one 10 minute bout of MVPA. A study investigating German adults aged between 65–89 years reported a similar tendency, showing a stronger association between disability and PA than between multimorbidity and PA [16].

No stable association of hypertension, asthma, diabetes, COPD, walking difficulties, pain/physical complaints, or anxiety/depression with time spent in MVPA was found. This result should be interpreted with caution due to the low prevalence of morbidities such as COPD (9%) or diabetes (7%) and probably mainly mild disease stages in our study population. Despite the possibility that more active subjects participated in the PA assessment, our results indicate that there is an urgent need to improve PA among adults in southern Germany, given that subjects spent a median of 61% of the time sedentary, and in contrast only 3% in MVPA/day. Furthermore, only 14% met the WHO PA recommendation.

The present results, including subjects aged 48–68 years resident in the region of Augsburg in southern Germany, are limited to the selected study population which had a relatively low response of 54%, likely leading to a potential risk of selection bias. Further, our results are based on cross-sectional data, which does not allow generalizations about long term PA behavior. Within our study population, the prevalence of morbidities was low and probably at early stages. Furthermore, these were assessed by questionnaires only and were not verified by an examination from a physician. The assessment of pain or physical complaints, and walking difficulties was based on self-report without precise definition of the cause.

Despite these limitations, this study has several strengths revealing new insights into PA levels and duration pattern of German adults assessed by accelerometry. Data on objectively measured PA among German adults is rare. Despite the inability to measure all PA engagement equally well, the advantage of the use of accelerometers is the ability to analyze the intensity and duration of consecutive minutes of PA, while minimizing the risk of reporting bias occurring in self-reports [5, 6, 9, 10].

There is a lot of methodological diversity among studies investigating PA with accelerometers [22]. This is partly due to the use of different devices, the investigation of uni- or triaxial movement measures as well as the chosen cut-points [11, 21–23]. Thus we provided data based on different approaches to illustrate the potential impact on the measured PA of adults in our study and to provide data for comparability with other studies published.

## Conclusions

In conclusion, the prevalence of being physically active was found to be generally low among adults from southern Germany. Overall, time spent in MVPA was accumulated mainly in very short bouts and one third of the population did not achieve a single 10 minute bout of MVPA contributing towards the WHO PA recommendation. Furthermore, only one in eight subjects achieved the WHO threshold, indicating a further need for PA promotion in Germany, particularly among older and obese subjects.

## Supporting information

S1 Fig Time spent in moderate to vigorous activity (MVPA) (triaxial cut-offs) in at least 1, 5, and 10 minute bouts categorized by age and adherence to WHO physical activity recommendations. (PDF)

S1 Methods Detailed description of diary, quality control and data imputation. (PDF)

S1 Tables Tables A-G. (PDF)
