# Supplementary material for: Physical activity levels, duration pattern and adherence to WHO recommendations in German adults
Source: PLoS One. 2017 Feb 28;12(2):e0172503. doi: 10.1371/journal.pone.0172503 (PMC5330478; doi:10.1371/journal.pone.0172503)
Supplement: S1 Fig — (PDF) [file pone.0172503.s001.pdf]

S1 Fig. Time spent in moderate to vigorous activity (MVPA) (triaxial cut-offs) in at least 1, 5, and 10 minute bouts categorized by age and adherence to WHO physical activity recommendations.

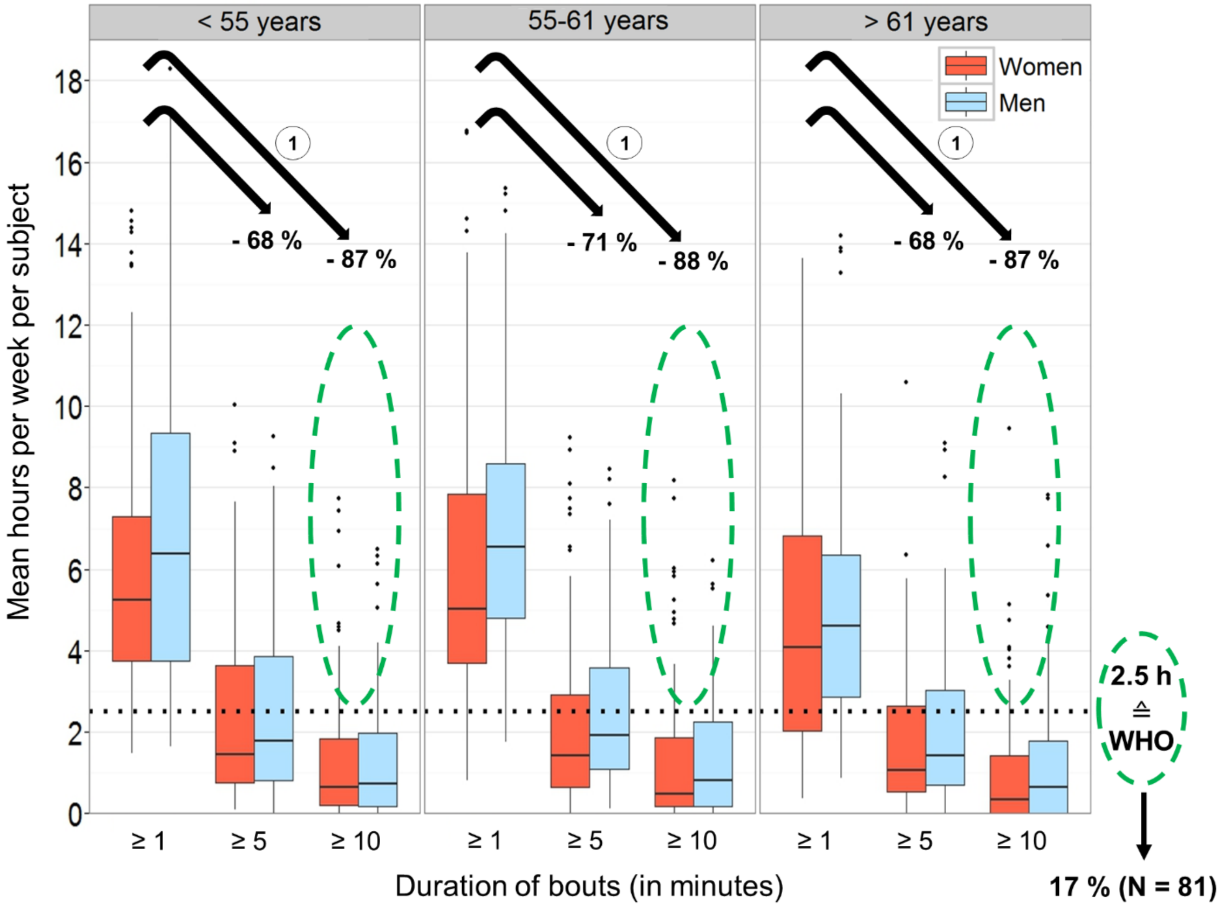

Hours/day spent in MVPA are averaged over the recording period of each subject and multiplied by seven. MVPA cut-off set at 2690 counts/minute (triaxial). - - - Subjects who met the WHO physical activity recommendation of ≥ 2.5 hours of MVPA/week in at least 10 minute bouts.

① Median percent decrease in time spent in MVPA after excluding bouts shorter than 5 and 10 minutes, respectively.
