# Supplementary material for: Physical activity levels, duration pattern and adherence to WHO recommendations in German adults
Source: PLoS One. 2017 Feb 28;12(2):e0172503. doi: 10.1371/journal.pone.0172503 (PMC5330478; doi:10.1371/journal.pone.0172503)
Supplement: S1 Methods — (PDF) [file pone.0172503.s002.pdf]

S1 Methods: Detailed description of diary, quality control and data imputation.

Diary

Participants were asked to fill out a detailed course of the day diary for seven consecutive days. For a better understanding, a one day example was provided. The diary was provided in German language to the participants and was translated to English for publication only.

Course of the day	Example
**Date**	**13.04.2012**
**Getting up: Sensor from wrist to hip (time)**	6:15
**How well did you sleep? (1: Very good to 6: Poor)**	3
How do you get to work?<br>(e. g. by car, bus)	Car
Time you leave the house - to work	7:30
Starting time of work	8:00
End time of work	18:00
Arrival time at home	18:45
Have you taken a nap?<br>(if yes, time from … to)	13:00 -<br>13:45
Type of physical activity e.g. soccer, basketball, hiking, cycling<br>Was this activity performed as member of a sports club or gym?<br>Start ……………<br>Finish …………..	Soccer<br><br>Yes<br>19:30<br>21:15
**Going to bed time**	23:30
**How did you feel today?** →	2
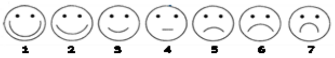	
Time and reason for removing the accelerometer	
	Example
Time and duration	6:20 - 6:40
Reason	Showering
Time and duration	19:45 -<br>21:00
Reason	Swimming

Quality control

We performed several steps to assure the best possible data quality. The number of non-valid days was 635 (16.4%). Wearing days were excluded if one or more of the below described exclusion criteria were met. Days were excluded if:

- differences occurred by comparison of the wear time between the diary report and the accelerometric data. Therefore, an algorithm based on the algorithm derived from the National Health and Nutrition Examination Survey (http://riskfactor.cancer.gov/tools/nhanes_pam) was applied. Non-wear time periods were defined as at least 60-minute time intervals of consecutive zero counts, allowing up to two consecutive minutes with nonzero counts less than or equal to 100 counts. Differences between this non-wear time algorithm and the reported wear time were calculated in minutes for each event. Because of the uncertainty of misclassification of wear periods as non-wear time and the other way round, we based our limits for exclusion of time intervals near the 3th and 97th percentile of the difference distribution. The limits were set to > |- 120| min difference between algorithm and diary, if algorithm reported non-wear time, and > 60 minutes, if the subject reported non-wear time, but the accelerometer showed counts of wear time → 317 days
- time of getting up or going to sleep could not be assessed or were missing > 4 times throughout the measuring period → 212 days
- the wear time was < 10 hours according to the diary (exception: if the total time spent awake was only between 8 to 10 hours than this day was excluded if the reported wear time in the diary was < 7 hours) → 74 days
- non wear-time was not at all reported through the whole wear time period, despite the fact that the accelerometer is not waterproof → 71 days
- reported sport activities without wearing the accelerometer lasted > 2 hours, e.g. during a competition or skiing → 43 days
- the sensor was defective or worn incorrectly (upside down or on hand instead of hip) → 13 days
- the subject reported to be sick (excluding headache or cold) on 2 or more days in a row (exclusion of days from the second day on) → 13 days
- the subject reported to be sick on most of the reporting days → 10 days
- the subject reported to be on a trip – meaning non-regular hole day activity - on 2 or more days in a row (exclusion of days from the second day on) → 1 day

Imputation of non-wear time periods during sport

To account for MVPA obtained through sport activities lasting < 2 hours when the accelerometer was not worn, e.g. during swimming or material arts, we imputed non-wear time MVPA. If the reported non-wear time period lasted between 30 and 120 minutes, we subtracted 15 minutes before imputation accounting for preparing for PA, e.g. changing clothes. The efficiency (e.g. % of time spent in MVPA during PA) of each subject as well as sex specific averages were calculated. If the subject had a period of reported PA while wearing the accelerometer, MVPA of non-wear time sport was imputed through multiplication of time spent in PA without wearing the accelerometer by his or her efficiency and added to the subjects total MVPA on this particular day. If no PA activities were reported where the accelerometer was worn, sex specific efficiency averages were used for imputation.
