# Supplementary material for: Physical activity levels, duration pattern and adherence to WHO recommendations in German adults
Source: PLoS One. 2017 Feb 28;12(2):e0172503. doi: 10.1371/journal.pone.0172503 (PMC5330478; doi:10.1371/journal.pone.0172503)
Supplement: S1 Tables — (PDF) [file pone.0172503.s003.pdf]

S1 Tables: Tables A-G.

Table A. Comparison of subjects participating in accelerometry compared to all other participants designated to participate in the "Lung health & physical activity" section of the KORA FF4 follow-up (n/N (%)).

**Participating in accelerometry**	**Yes** (N=562)	**No** (N=481)
Sex, male	264/562 (47)	224/481 (47)
Age, years		
< 55	189/562 (34)	145/481 (30)
55-61	201/562 (36)	159/481 (33)
> 61	172/562 (31)	177/481 (37)
Working, yes	400/561 (71)	317/481 (66)
Education		
Low (< 10 years of school)	261/561 (47)	238/479 (50)
Medium (= 10 years of school)	153/561 (27)	117/479 (24)
High (> 10 years of school)	147/561 (26)	124/479 (26)
Body mass index, kg/m^2		
Normal (< 25)	156/562 (28)	154/481 (32)
Overweight (< 30)	245/562 (44)	181/481 (38)
Obese (≥ 30)	161/562 (29)	146/481 (30)
Overall health (reported)*		
Excellent/very good	155/562 (28)	102/479 (21)
Good	346/562 (62)	304/479 (63)
Fair/poor	61/562 (11)	73/479 (15)
Hypertension, yes	185/561 (33)	184/481 (38)
Diabetes, yes	37/561 (7)	37/481 (8)
Asthma ever, yes	54/562 (10)	47/481 (10)
COPD, yes	48/562 (9)	34/480 (7)
Myocardial infarction, yes	17/561 (3)	14/481 (3)
Stroke, yes	12/562 (2)	11/477 (2)
Difficulties in walking		
Not at all/slight	523/559 (94)	439/479 (92)
Moderate/hard	36/559 (6)	40/479 (8)
Pain or physical complaints		
Not at all/slight	476/562 (85)	388/479 (81)
Moderate/hard	86/562 (15)	91/479 (19)

Participating in accelerometry	Yes (N=562)	No (N=481)
Feeling anxious/depressed		
Not at all/slight	519/560 (93)	439/478 (92)
Moderate/strong	41/560 (7)	39/478 (8)

*p < 0.05 in Chi-square test. COPD: Chronic obstructive pulmonary disease.

Table B. Median time spent in the three activity levels – sedentary, light and moderate to vigorous (MVPA) stratified by sex.

	**Uniaxial**		**Triaxial**	
	Males<br>47.2 % (224)	Females<br>52.8 % (251)	Males<br>47.2 % (224)	Females<br>52.8 % (251)
**Median (25th, 75th percentile) of minutes per day averaged per subject**				
**Sedentary**	586 (520, 646)*	529 (473, 587)	504 (434, 577)*	435 (376, 498)
**Light**	303 (253, 369)*	343 (291, 396)	370 (308, 437)*	416 (362, 471)
**MVPA**	35 (21, 49)*	28 (17, 47)	49 (33, 71)*	43 (28, 62)
**Median % (25th, 75th percentile) of recorded time spent in each level per day averaged per subject**				
**Sedentary**	63 (56, 69)*	58 (52, 65)	54 (47, 62)*	48 (42, 55)
**Light**	32 (27, 40)*	38 (33, 44)	40 (34, 48)*	46 (40, 52)
**MVPA**	4 (2, 5)*	3 (2, 5)	5 (4, 8)	5 (3, 7)

*$p < 0.05$ in Wilcoxon rank-sum test (males vs. females).

Table C. Median time spent in moderate to vigorous activity stratified either by age tertile or body mass index (BMI) for males and females.

		Uniaxial		Triaxial	
		Males	Females	Males	Females
**Median (25th, 75th percentile) of minutes per day averaged per subject**					
**Age**, years	< 55	35 (24, 57)[1]	31 (21, 52)[1]	57 (32, 80)[1]	45 (32, 65)[1]
	55 - 61	40 (29, 54)[2]	28 (17, 46)	56 (41, 74)[2]	43 (32, 67)
	> 61	26 (15, 43)	21 (10, 42)	39 (26, 55)	38 (17, 58)
**BMI,** kg/m^2	Normal (< 25)	44 (30, 59)[3]	34 (20, 54)[3]	55 (39, 79)	50 (30, 72)[3]
	Overweight (≥ 25)	36 (21, 51)[4]	28 (18, 40)[4]	49 (32, 70)	40 (32, 58)
	Obese (≥ 30)	28 (17, 41)	20 (11, 33)	43 (32, 63)	35 (21, 57)

Significant difference (p < 0.05) by pairwise Wilcoxon rank-sum test stratified by sex between the categories:

[1] age < 55 and age > 61

[2] age 55-61 and age > 61

[3] normal and obese

[4] overweight and obese

Table D. Bout-length of median hours/week spent in moderate to vigorous activity stratified by sex and age.

		Uniaxial		Triaxial	
		Males	Females	Males	Females
**Median hours (25th, 75th percentile) of MVPA/week**					
**A**	≥ 1 min bouts	4.00 (2.43, 5.68)*	3.22 (1.92, 5.4)	5.76 (3.78, 8.12)*	5.02 (3.22, 7.21)
	≥ 5 min bouts	1.27 (0.53, 2.95)	0.99 (0.35, 2.45)	1.75 (0.83, 3.42)	1.38 (0.64, 2.98)
	≥ 10 min bouts	0.59 (0, 1.75)	0.35 (0, 1.36)	0.70 (0.17, 2.01)	0.52 (0, 1.76)
**Median % (25th, 75th percentile) of MVPA/week spent in ≥ 5 and ≥ 10 minute bouts of total MVPA/week**					
**B**	**≥ 5 min bouts**				
	Age, years				
	< 55	32 (20, 49)	39 (17, 55)	32 (21, 42)	32 (19, 46)
	55 - 61	30 (19, 55)	34 (17, 54)	29 (20, 50)	28 (17, 46)
	> 61	37 (15, 59)	34 (12, 54)	33 (15, 49)	32 (16, 51)
	**≥ 10 min bouts**				
	Age, years				
	< 55	13 (0, 33)	12 (0, 38)	14 (2, 25)	13 (4, 37)
	55 - 61	15 (0, 34)	17 (0, 31)	11 (4, 29)	12 (4, 28)
	> 61	17 (0, 37)	9 (0, 32)	16 (0, 30)	10 (0, 32)
**% (n) reached WHO recommendation**					
		16.1 (36)	12.0 (30)	18.3 (41)	15.9 (40)

*p < 0.05 (pairwise) Wilcoxon rank-sum test between (A) males and females, and (B) age categories stratified by sex.

Table E. Achieved bout-length applying uniaxial cut-offs.

% (N) achieved bout of MVPA	< 10 minutes[1] (N=168)	≥ 10 minutes[2] (N=241)	≥ 10 minutes + adherence to WHO[3] (N=66)
Sex			
Male	32.1 (72)	51.8 (116)	16.1 (36)
Female	38.2 (96)	49.8 (125)	12.0 (30)
Age, years (range: 48 - 68)			
< 55	32.9 (51)	52.3 (81)	14.8 (23)
55-61	31.5 (53)	52.4 (88)	16.1 (27)
> 61	42.1 (64)	47.4 (72)	10.5 (16)
Body mass index, kg/m^2 *			
Normal (< 25)	25.5 (35)	50.4 (69)	24.1 (33)
Overweight (≥ 25)	35.5 (72)	53.7 (109)	10.8 (22)
Obese (≥ 30)	45.2 (61)	46.7 (63)	8.1 (11)
Education*			
Low (< 10 years of school)	45.4 (99)	45.9 (100)	8.7 (19)
Medium (= 10 years of school)	32.6 (45)	48.6 (67)	18.8 (26)
High (> 10 years of school)	20.2 (24)	62.2 (74)	17.6 (21)
Hypertension*			
No	30.3 (95)	53.8 (169)	15.9 (50)
Yes	45.3 (73)	44.7 (72)	9.9 (16)
Diabetes			
No	34.5 (153)	51.5 (228)	14.0 (62)
Yes	46.9 (15)	40.6 (13)	12.5 (4)
Asthma			
No	34.1 (146)	51.4 (220)	14.5 (62)
Yes	46.8 (22)	44.7 (21)	8.5 (4)
COPD			
No	34.6 (150)	51.5 (223)	13.9 (60)
Yes	42.9 (18)	42.9 (18)	14.3 (6)
Difficulties in walking*			
Not at all/slight	33.7 (150)	52.1 (232)	14.2 (63)
Moderate/hard	60.7 (17)	32.1 (9)	7.1 (2)
Pain or physical complaints*			
Not at all/slight	31.5 (128)	53.2 (216)	15.3 (62)
Moderate/hard	58.0 (40)	36.2 (25)	5.8 (4)
Feeling anxious/depressed			
Not at all/slight	34.6 (153)	52.0 (230)	13.3 (59)
Moderate/strong	43.8 (14)	34.4 (11)	21.9 (7)

[1]subjects who did not achieve moderate to vigorous activity (MVPA) in bouts of at least 10 minutes. [2]subjects who achieved bouts of at least 10 minutes, but less than 2.5 hours per week. [3]subjects who met the WHO recommendation of 2.5 hours a week in bouts of at least 10 minutes. *$p < 0.05$ Chi-square test or Fisher's Exact Test (if cell counts < 5). COPD: Chronic obstructive pulmonary disease.

Table F. Count ratios (95% confidence intervals) of triaxial average minutes per day of moderate-to-vigorous physical activity (MVPA), sedentary or light activity estimated by negative binomial regression.

**Outcome**		**Sedentary**		**Light**		**MVPA**	
Variables	Model	Basic[1]	Stepwise[1]	Basic[1]	Stepwise[1]	Basic	Stepwise
Sex	Female	-	-	-	-	-	-
	Male	**1.09 (1.05; 1.13)**	**1.09 (1.05; 1.13)**	**0.89 (0.85; 0.92)**	**0.89 (0.85; 0.92)**	**1.18 (1.06; 1.31)**	**1.18 (1.06; 1.31)**
Age	< 55	-	-	-	-	-	-
	55-61	0.96 (0.92; 1.00)	**0.95 (0.91; 0.99)**	1.05 (1.00; 1.10)	**1.05 (1.01; 1.10)**	1.01 (0.89; 1.15)	1.02 (0.90; 1.15)
	> 61	1.03 (0.98; 1.08)	1.04 (0.99; 1.08)	0.99 (0.94; 1.04)	0.99 (0.94; 1.04)	**0.77 (0.67; 0.87)**	**0.76 (0.67; 0.87)**
BMI	Normal	-	-	-	-	-	-
	Overweight	1.04 (0.99; 1.08)	**1.05 (1.00; 1.09)**	0.97 (0.93; 1.02)	0.96 (0.92; 1.01)	**0.88 (0.78; 1.00)**	**0.88 (0.77; 0.99)**
	Obese	**1.11 (1.05; 1.16)**	**1.12 (1.07; 1.18)**	**0.91 (0.86; 0.96)**	**0.90 (0.85; 0.95)**	**0.79 (0.69; 0.91)**	**0.78 (0.68; 0.90)**
Education	Low	-	-	-	-	-	-
	Medium	-	**1.07 (1.02; 1.11)**	-	**0.93 (0.89; 0.98)**	-	0.97 (0.86; 1.10)
	High	-	**1.12 (1.07; 1.17)**	-	**0.90 (0.86; 0.94)**	-	**0.85 (0.75; 0.97)**
Pain/physical complaints	Not at all/slight	-	-	-	-	-	-
	Moderate/hard	-	x	-	x	-	0.87 (0.75; 1.01)
Diabetes	No	-	-	-	-	-	-
	Yes	-	**1.10 (1.02; 1.18)** [stratified by sex: **1.18 (1.07; 1.30)** females; 0.99 (0.89; 1.11) males]	-	**0.90 (0.83; 0.97)** [stratified by sex: **0.81 (0.74; 0.89)** females; 1.02 (0.90; 1.16) males]	-	x

Significant associations ($p < 0.05$) are shown in bold. Basic model was adjusted for sex, age, and body mass index (BMI). Besides sex, age, and BMI considered variables in the stepwise selection model were: season, education, hypertension, diabetes, asthma, chronic obstructive pulmonary disease (COPD), difficulties with walking, pain or physical complaints, and anxiety/depression. Models with the lowest Akaike information criterion (AIC) are shown. [1]Model additionally adjusted for average recorded wear time/day. [x]Covariate did not remain in main model.

Table G. Achieved bout-length applying triaxial cut-offs.

% (N) achieved bout of MVPA	< 10 minutes[1] (N=116)	≥ 10 minutes[2] (N=278)	≥ 10 minutes + adherence to WHO[3] (N=81)
Sex			
Male	23.2 (52)	58.5 (131)	18.3 (41)
Female	25.5 (64)	58.6 (147)	15.9 (40)
Age, years (range: 48 - 68)			
< 55	23.9 (37)	58.1 (90)	18.1 (28)
55-61	19.6 (33)	61.3 (103)	19.0 (32)
> 61	30.3 (46)	55.9 (85)	13.8 (21)
Body mass index, kg/m^2 *			
Normal (< 25)	17.5 (24)	54.7 (75)	27.7 (38)
Overweight (≥ 25)	26.6 (54)	59.6 (121)	13.8 (28)
Obese (≥ 30)	28.1 (38)	60.7 (82)	11.1 (15)
Education*			
Low (< 10 years of school)	29.8 (65)	57.3 (125)	12.8 (28)
Medium (= 10 years of school)	24.6 (34)	53.6 (74)	21.7 (30)
High (> 10 years of school)	14.3 (17)	66.4 (79)	19.3 (23)
Hypertension,			
No	22.3 (70)	58.3 (183)	19.4 (61)
Yes	28.6 (46)	59.0 (95)	12.4 (20)
Diabetes,			
No	23.3 (103)	59.6 (264)	17.2 (76)
Yes	40.6 (13)	43.8 (14)	15.6 (5)
Asthma,			
No	23.4 (100)	59.1 (253)	17.5 (75)
Yes	34.0 (16)	53.2 (25)	12.8 (6)
COPD,			
No	23.6 (102)	59.4 (257)	17.1 (74)
Yes	33.3 (14)	50.0 (21)	16.7 (7)
Difficulties in walking*			
Not at all/slight	22.9 (102)	59.6 (265)	17.5 (78)
Moderate/hard	50.0 (14)	42.9 (12)	7.1 (2)
Pain or physical complaints*			
Not at all/slight	22.4 (91)	58.6 (238)	19.0 (77)
Moderate/hard	36.2 (25)	58.0 (40)	5.8 (4)
Feeling anxious/depressed			
Not at all/slight	24.2 (107)	59 (261)	16.7 (74)
Moderate/strong	25.0 (8)	53.1 (17)	21.9 (7)

[1]subjects who did not achieve moderate to vigorous activity (MVPA) in bouts of at least 10 minutes. [2]subjects who achieved bouts of at least 10 minutes, but less than 2.5 hours per week. [3]subjects who met the WHO recommendation of 2.5 hours a week in bouts of at least 10 minutes. *$p < 0.05$ Chi-square test or Fisher's Exact Test (if cell counts < 5). COPD: Chronic obstructive pulmonary disease.
